# Supplementary material for: Depressive and Anxiety Disorders of Parents of Children with Cancer
Source: J Clin Med. 2022 Sep 26;11(19):5670. doi: 10.3390/jcm11195670 (PMC9572351; doi:10.3390/jcm11195670)

Attachments

Questionnaire S1. Questionnaire supplementing the assessment of parents' mental health- authors own questionnaire.

Questionnaire S2. A questionnaire on complications in children after chemotherapy- authors own questionnaire

Questionnaire S3. A Visual Scale assessing complications in children after chemotherapy- authors own questionnaire

Questionnaire S1. Questionnaire supplementing the assessment of parents' mental health- authors own questionnaire.

Questionnaire supplementing the assessment of parents' mental health

I would like to kindly ask you to fill in an anonymous questionnaire that will allow me to measure your mental health. The obtained data will allow me to publish the results for publication entitled "Depressive and anxiety disorders of parents of children with cancer".

The questions concern the period from the diagnosis of childhood cancer.

Please mark the correct answer with a cross.

Thank you

1. I go out to meet my family and friends less often:
   - Yes
   - No
   - I don't know
2. I show less interest in other people's problems (I do not listen about their issues; I do not offer my help):
   - Yes
   - No
   - I don't know
3. Other people often irritate me (I lose my patience easily):
   - Yes
   - No
   - I don't know
4. I show less emotions than before the child's disease:
   - Yes
   - No
   - I don't know
5. I have less contact with people:

- Yes
- No
- I don’t know

6. My relationship with my immediate family/friends has deteriorated:
- Yes
- No
- I don’t know

7. I pay less attention to my appearance:
- Yes
- No
- I don’t know

8. I cannot concentrate for a long time:
- Yes
- No
- I don’t know

9. I work less or no hours:
- Yes
- No
- I don’t know

10. I am less effective at work:
- Yes
- No
- I don’t know

11. I leave the house less often for entertainment purposes:
- Yes
- No
- I don’t know

12. I spend less time on my passions and hobbies:
- Yes
- No
- I don’t know

13. I completely gave up active leisure (sport, walking etc.):
- Yes
- No
- I don’t know

14. I still enjoy an active life:
- Yes
- No
- I don’t know

Questionnaire S2. A questionnaire on complications in children after chemotherapy- authors own questionnaire

A questionnaire on complications in children after chemotherapy- authors own questionnaire

I would like to ask you to fill in an anonymous questionnaire, the purpose of which is to investigate the occurrence of complications in children after chemotherapy. The survey should be completed by the child's caretaker (parent). The obtained data will allow me to develop the results for publication entitled "Depressive and anxiety disorders of parents of children with cancer". Please mark a correct answer with a cross. Multiple-choice questions may include multiple answers.

Thank you.

1. The gender of a child

   ☐ A girl

   ☐ A boy

2. The age of a child

   Please type: …………………….

3. The type of diagnosed cancer

   Please type: ……………………………………

4. At what age was the cancer diagnosed?

   Please type: ………………………………

5. Duration of the oncological disease"

   ☐ Up to 6 months

   ☐ 7-12 months

   ☐ 13-24 months

   ☐ Over 24 months

6. Has anyone in the immediate family suffered from cancer? If so, please tick (multiple choice):

   ☐ Mother

- ☐ Father
- ☐ Brother
- ☐ Sister
- ☐ Grandmother
- ☐ Grandfather
- ☐ No one in the family suffered from cancer

7. Have been any complications in child after chemotherapy:
   - ☐ yes
   - ☐ no
8. If you answered yes in question 7, please specify which complications occurred after chemotherapy (multiple choice):

- ☐ Decreased appetite
- ☐ Weakness
- ☐ Dizziness
- ☐ Heart palpitations
- ☐ Easy fatigability
- ☐ Pale skin
- ☐ Nose or gum bleading
- ☐ Oral lesions (eg. erosions)
- ☐ Sialorrhea
- ☐ Pain in the oral cavity
- ☐ Difficulty swallowing
- ☐ Swelling of the face or neck
- ☐ Diarrhoea
- ☐ Nausea and vomiting
- ☐ Fever
- ☐ Abdominal pain
- ☐ Hair loss
- ☐ Skin discolouration
- ☐ Cough
- ☐ Hemoptysis
- ☐ Difficulty breathing
- ☐ Other (please specify): ……………………………

Questionnaire S3. A Visual Scale assessing complications in children after chemotherapy- authors own questionnaire

A Visual Scale assessing complications in children after chemotherapy- authors own questionnaire

I would like to kindly ask you to fill in an anonymous questionnaire, investigating the occurrence of complications after chemotherapy in children undergoing oncology treatment and the severity of complications. The survey should be completed by the child's caretaker (parent). The obtained data will allow me to develop the results for publication entitled "Depressive and anxiety disorders of parents of children with cancer".

Please mark a correct answer on a 0-10 scale.

Thank you.

On a scale 0-10, please mark, how inconvenient for child was given symptom:

0- no symptom
2- very rarely
4- a few times
6- often
8- almost all the time, the symptom significantly lowered the child's mood
10- intolerable, significantly inhibited everyday functioning

1. Decreased appetite

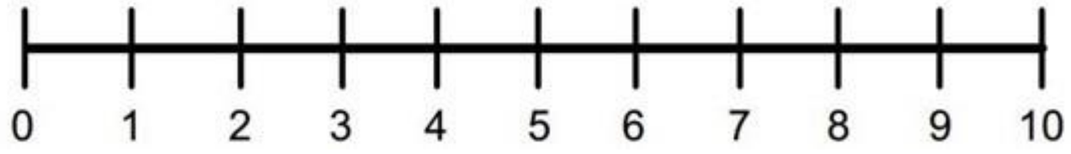

2. Weakness

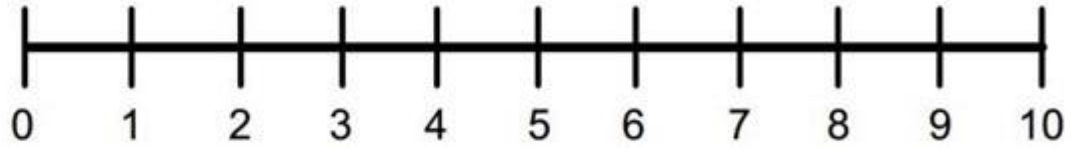

3.Headache

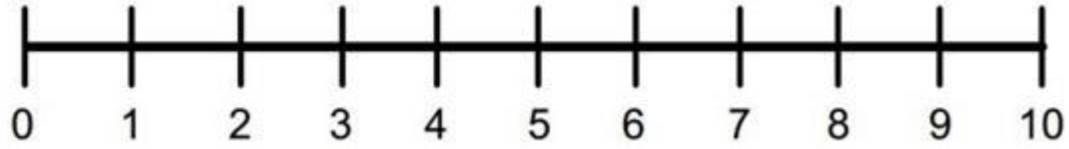

4. Dizziness

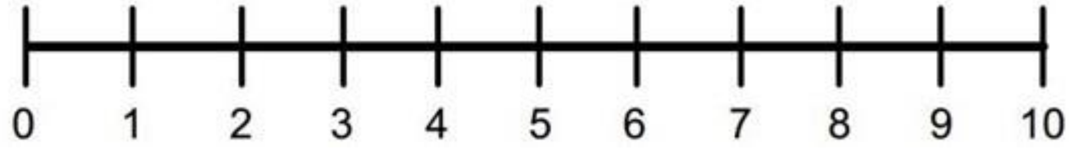

5. Pain in the oral cavity

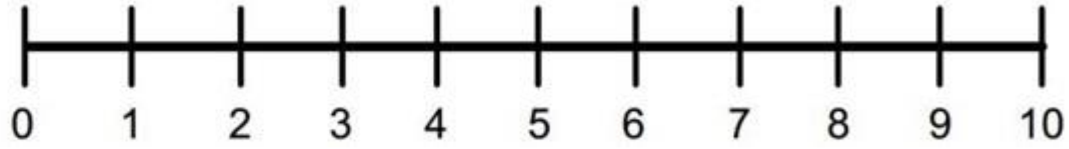

6. Diarrhoea

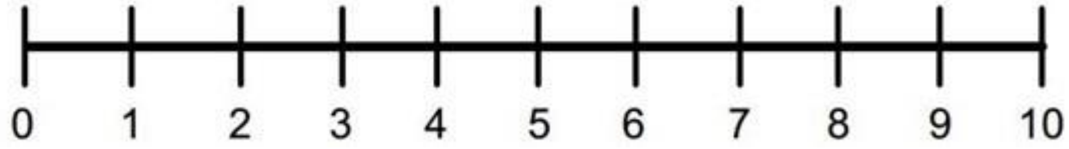

7. Constipation

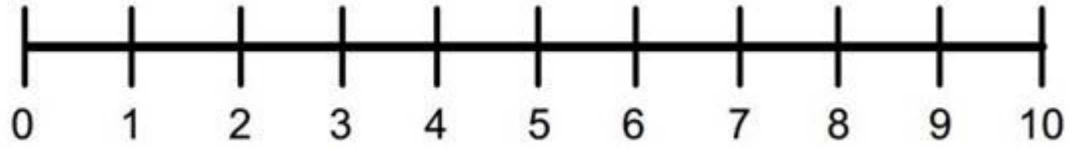

8. Abdominal pain

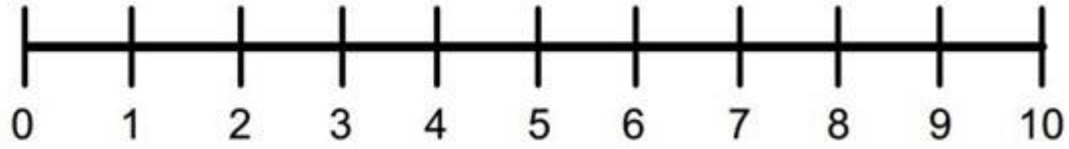

9. Nausea and vomiting

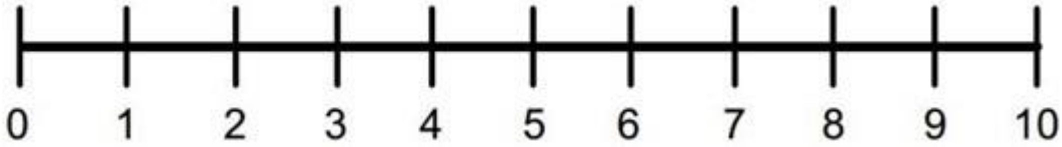

10. Fever

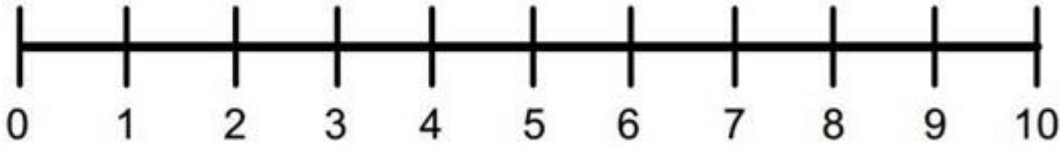

Supplement: Supplementary file 1 [file jcm-11-05670-s001.zip › jcm-1882217-supplementary.pdf]
